# Treatment of Hematomas Using a Synthetic Hybrid-Scale Fiber Matrix

**DOI:** 10.7759/cureus.26491

**Published:** 2022-07-01

**Authors:** Eric W Temple

**Affiliations:** 1 Foot and Ankle Surgeon, The Iowa Clinic, West Des Moines, USA

**Keywords:** synthetic hybrid-scale fiber matrix, ­wound healing, skin substitute, subcutaneous hematoma, hematoma evacuation, trauma

## Abstract

Effective treatment of traumatic subcutaneous hematomas is important to avoid complications, including skin necrosis, infection, scarring, hyperpigmentation, tissue edema, and prolonged recovery. Hematoma treatment may include evacuation followed by application of a skin substitute. Given the challenges associated with conventional skin substitutes, a fully-synthetic, resorbable, electrospun matrix composed of hybrid-scale fibers may offer a new option for treating hematomas. The present study reports on two clinical case reports assessing the use of the synthetic hybrid-scale fiber matrix for the treatment of hematomas.

The hematomas located on the pretibial leg and dorsal foot were debrided in the operating room with evacuation of the hematomas, and the synthetic matrix was applied to the wounds. Following treatment, the wounds were observed for healing progress, including measuring and photographing the wounds and documenting clinical observations. The synthetic hybrid-scale fiber matrix was re-applied as needed based on clinician discretion.

In both cases, treatment following the use of the synthetic hybrid-scale fiber matrix resulted in complete healing. Complete closure of all wounds was observed after two to three applications of the synthetic matrix within six to 16 weeks, and no adverse events were noted.

In this study, hematomas of the foot and the leg demonstrated successful healing following treatment with the synthetic hybrid-scale fiber matrix. The successful clinical outcomes suggest that this biomaterial may offer benefits as part of a new treatment paradigm for hematomas and warrants further investigation.

## Introduction

Subcutaneous hematomas can result from trauma. At particular risk are elderly patients with multiple co-morbidities and those who may be taking medications such as anticoagulants [1-3]. Effective treatment of subcutaneous hematomas is important in order to avoid complications including skin necrosis, infection, scarring, hyperpigmentation, tissue edema, and prolonged recovery [1-5]. Hematoma evacuation may be done using a surgical approach, in which the hematoma is evacuated under general or regional anesthesia followed by treatment with a skin graft or skin substitute [3,5]. There are several traditional dermal treatment options, including autografts, allografts, and xenografts. However, these modalities are associated with limited availability and donor site morbidity in the case of autografts, as well as potential disease transmission and immunologic rejection in the case of allografts and xenografts [6].

Given the challenges associated with conventional treatment options, a fully-synthetic, resorbable, electrospun matrix composed of hybrid-scale fibers may offer a new option for treating hematomas [7]. The biomaterial is engineered with a microarchitecture similar to the extracellular matrix (ECM) of native skin, supporting cellular infiltration, neovascularization, and new tissue formation [7]. The synthetic hybrid-scale fiber matrix gradually resorbs over an average of a few weeks at a rate designed to align with the rate of new tissue formation [7]. The synthetic skin substitute does not carry the risks of disease transmission or inflammatory response associated with biologic materials [7]. The synthetic matrix is composed of two biocompatible, bioresorbable polymers, polydioxanone and polyglactin 910, which are electrospun to form a soft, durable, nonfriable sheet. This can be stored at room temperature until ready to use, at which time it can be fenestrated, cut to size, and secured using the clinician’s preferred method [7]. Preclinical assessment in a porcine wound healing model found that the synthetic hybrid-scale fiber matrix demonstrated superior healing quality and an accelerated rate of healing compared to a bilayer xenograft matrix, including faster collagen deposition, neovascularization, granulation tissue formation, and wound closure [8]. Clinical application of the synthetic matrix has also demonstrated significant wound healing across multiple wound types, including recalcitrant foot ulcers, venous leg ulcers, diabetic foot ulcers, and pressure ulcers [9-12].

The aim of the present two case studies was to evaluate the use of the synthetic hybrid-scale fiber matrix for the treatment of hematomas following debridement and evacuation.

## Case presentation

Case One

An 82-year-old Caucasian male had been wearing compression socks continuously for a week when he dropped a heavy item on his foot, resulting in a hematoma. The patient had multiple co-morbidities, including a bleeding disorder, chronic left foot ulcer proceeded by the hematoma, diabetes mellitus with neuropathy, lymphedema, nephrolithiasis, and morbid obesity. The patient was on Xeralto (rivaroxaban, Janssen Pharmaceuticals, Titusville, New Jersey) for his bleeding disorder, exacerbating the hematoma.

**Figure 1:**
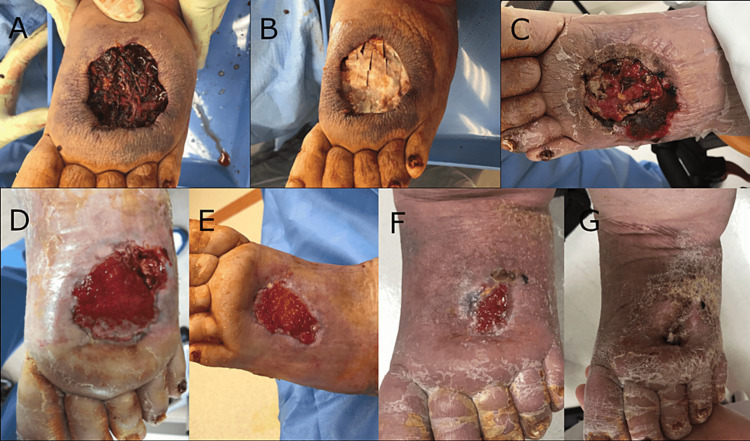
Foot hematoma treated using the synthetic hybrid-scale fiber matrix. Wound (a) at baseline, (b) at initial application of matrix, (c) two weeks after initial application, (d) five weeks after initial application, (e) eight weeks after initial application, (f) 12 weeks after initial application, and (g) completely healed 16 weeks after initial application.

Upon presentation, the wound size measured 6.5 cm x 5.8 cm with necrotic eschar and ischemic wound edges (Figure 1A). The patient was taken to the operating room (OR), where the wound was debrided, evacuating the hematoma and resulting in a wound size of 6.7 cm x 5.9 cm x 4.9 cm (Figure 1B). The synthetic hybrid-scale fiber matrix was fenestrated and applied to the wound bed, followed by the application of negative pressure wound therapy (NPWT). NPWT was changed three times on Monday, Wednesday, and Friday. One week later, the patient was seen for NPWT change, at which time the synthetic matrix was observed to be intact, and continuous NPWT was reapplied at 85 mmHg (Figure 1C). The NPWT pressure was elected to avoid lifting the graft off the wound bed. One month following initial debridement, the patient was seen for NPWT change, and the wound was observed to have decreased to 4 cm x 5 cm x 0.1 cm with approximately 90% granular tissue and 10% fibrotic with some necrotic tissue (Figure 1D). Sharp debridement was performed. NPWT was stopped as the wound had achieved granulation. After another week, the wound measured 5.4 cm x 3.5 cm x 0.1 cm with a continued small amount of necrotic tissue. After another 12 days, the patient was taken back to the OR for debridement and re-application of the synthetic matrix secured with 3-0 nylon sutures, at which time the wound measured 4 cm x 2 cm x 0.1 cm (Figure 1E). At the two-week follow-up after the second debridement, the wound was observed to have continued to decrease in size to 3 cm x 2.3 cm x 0.1cm, with granulation tissue present. Profore wrap (Smith + Nephew, Fort Worth, Texas) was applied to help with the lymphedema. After another week, the wound measured 2.5 cm x 1.9 cm x 0.1 cm, the wound was debrided, and a third application of the synthetic hybrid-scale fiber matrix was applied in the office and sutured in place. A week after the third application of the synthetic matrix, the dressing was removed, and the matrix was observed to be incorporated with hypergranulation (Figure 1F). Silver nitrate was applied to the wound, and Profore wrap was applied. The patient missed the next two-week follow-up visit but had home health care continue with dressing changes. The patient was seen after another two weeks, 16 weeks after the initial application, and the wound was completely healed (Figure 1G). The patient was instructed to use compression socks to prevent further wound issues.

Case Two

**Figure 2:**
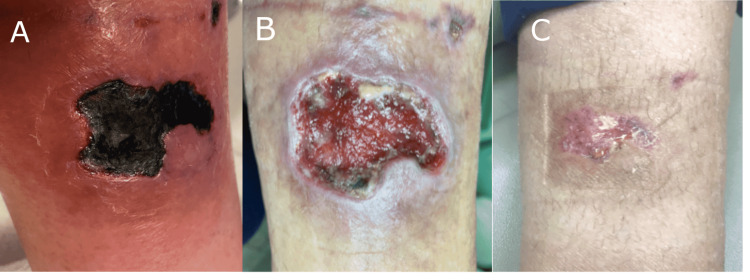
Leg hematoma treated using the synthetic hybrid-scale fiber matrix. Wound (a) at baseline, (b) with continued ischemia and necrotic tissue pre-debridement and prior to application of the synthetic matrix, and (c) completely healed 6 weeks after initial application of the synthetic matrix.

A 59-year-old Caucasian female presented initially to a wound nurse with a pretibial wound that had become necrotic with hematoma (Figure 2A). The patient had been seen and treated for the past four weeks by her primary care physician after having a fall and hitting her shin on metal stairs. The patient had multiple co-morbidities, including anxiety, vertigo, vertebral fracture, diverticulosis, hypothyroidism, obesity, chronic leg ulcer, and depression. The patient had been using wet-to-dry dressings, compression socks, and Silvadene. The patient was started on Santyl (Smith + Nephew, Fort Worth, Texas) and instructed to follow up with the surgeon. The patient was initially seen by the surgeon via an e-visit due to concern for coronavirus (COVID-19). At the e-visit, the wound was assessed to have no change, with continued ischemia to the wound edges and continued necrotic tissue with erythema to the periwound (Figure 2B). Due to COVID-19, surgery was delayed for two weeks. At the time of surgery, debridement with evacuation of the hematoma was performed, the wound measured 2.1 cm x 3.8 cm x.3 cm, and the synthetic hybrid-scale fiber matrix was applied. Three days post-surgery the dressings were changed, and new dressings with bolster were applied. One week post-surgery, the synthetic matrix was still intact in the wound bed, and new primary dressings were applied. Three weeks post-surgery the dressings were removed. The wound measured 1.1 cm x 0.8 cm x 0.1 cm and the synthetic matrix was observed to be incorporated into the wound. A second application of the synthetic matrix was utilized. Five weeks post-surgery, the wound was evaluated. The synthetic hybrid-scale fiber matrix was intact but dry, so wound gel was applied. Six weeks post-surgery and initial application, the dressings were removed, and the wound was observed to be completely healed (Figure 2C).

## Discussion

Two clinical case studies assessing the use of the synthetic hybrid-scale fiber matrix (Restrata®, Acera Surgical, St. Louis, Missouri) for the treatment of hematomas were conducted via retrospective review of patient charts. The study was exempt from institutional review board approval due to the absence of patient-identifying information. Patient charts were reviewed and data were collected regarding comorbidities and current medications. Each patient presented with a subcutaneous hematoma that formed as a result of traumatic injury to the lower extremity, one on the dorsal foot and one on the pretibial leg. The hematomas were evaluated by the operating physician and wound edges were assessed for viable tissue. The wounds were found to be necrotic and therefore required evacuation. 

The hematomas were debrided in the operating room with the evacuation of the hematomas with sharp debridement then flushed with normal sterile saline. A synthetic matrix was selected for these two patients over biologic grafts. This method was elected to avoid creating an additional wound on patients whose primary wounds were slow to heal and to avoid inflammatory responses and enzymatic breakdown often seen with biologic materials. The synthetic matrix was fenestrated with 15 blades and soaked in saline prior to application. The matrix was then applied to the wounds and sutured in place using 3-0 nylon and dressed in a non-adherent primary dressing, gauze, and bandaged for compression. Following treatment, the wounds were observed for healing progress, including measuring and photographing the wounds and documenting clinical observations. The patients returned at weekly and biweekly intervals post initial evacuation. The synthetic hybrid-scale fiber matrix was re-applied as determined by clinician discretion and evaluation of regranulation tissue. The initial application of the synthetic hybrid-scale fiber matrix was conducted in the operating room as the patients were inpatient at the time. The graft was re-applied in both operating room and clinician office settings.

Two patients with traumatic hematomas were included in this preliminary study. In both cases, treatment following the use of the synthetic hybrid-scale fiber matrix resulted in complete healing.

Evacuation is recommended in large volume and necrotic hematomas [13]. This, however, can create wound healing challenges in cases of older patients or patients with multiple co-morbidities [13]. Skin grafts are commonly used to facilitate healing post hematoma evacuation [3,5], however, these treatment modalities present their own sets of challenges, such as donor site morbidity in autografts and immunologic response in allografts or xenografts [6]. In this preliminary case series, the synthetic hybrid-scale fiber matrix was successfully utilized in two patients with multiple comorbidities and resulted in no complications or infections. The synthetic hybrid-scale fiber matrix was applied at the time of evacuation and reapplied as clinically indicated. Both patients had complete wound closure within six to 16 weeks after initial application.

The efficacy of the synthetic hybrid-scale fiber matrix observed here is similar to findings in clinical studies of other wound types treated with the synthetic matrix, including an 85% complete wound closure rate of chronic lower extremity wounds (68 of 82 wounds) at 12 weeks after initial application [9] and a 96% wound closure rate of lower extremity wounds (22 of 23 wounds) with an average time to heal of 96.1 days [11]. Additional clinical studies utilizing the synthetic hybrid-scale fiber matrix have resulted in 75% complete wound closure rate of recalcitrant neuropathic foot ulcers (three of four wounds) after 13 to 28 weeks [10] and a 75% wound closure rate of diabetic foot ulcers (18 of 24 wounds) with an average time to complete closure being 6.4 weeks [12].

Similar positive results in patients with multiple comorbidities have been observed in previous studies. 20 patients with chronic diabetic foot ulcers and venous leg ulcers were treated with the synthetic hybrid-scale fiber matrix. These patients had a total of 132 secondary comorbidities and 96% of wounds demonstrated complete closure at 244 days (venous leg ulcers) and 122 days (diabetic foot ulcers) despite the previous failure with other advanced wound therapies (Poster, Husain K, Treatment of Complex Lower Extremity Wounds Utilizing Synthetic Hybrid-Scale Fiber Matrix). Both patients in the current study had multiple comorbidities, including bleeding disorders, diabetes mellitus, chronic ulcers, and obesity. These comorbidities put both of the patients at high risk for poor wound healing post evacuation, however, both patients exhibited excellent healing after two to three applications of the synthetic hybrid-scale fiber matrix.

The positive clinical findings in this limited case series could be due in part to the design of the matrix. The synthetic hybrid-scale fiber matrix is engineered to be similar to the native human extracellular matrix, therefore encouraging cellular ingrowth and neovascularization [7]. Since split-thickness skin grafting is often used in wound healing post hematoma evacuation, the synthetic hybrid-scale fiber matrix offers an alternative to this approach, eliminating risks such as donor site morbidity and inflammatory response [7]. As a synthetic, the matrix is resistant to enzymatic degradation and therefore remains adherent to the wound bed, even in circumstances in which split-thickness skin grafts have failed [14].

In addition to its clinical utility, the synthetic hybrid-scale fiber matrix may also offer advantages in light of COVID-19. The biomaterial persists in the wound bed for an average of a few weeks and does not require weekly re-applications or physical removal of the synthetic skin substitute [7], which limits the amount of monitoring needed in the hospital or clinician's office and thus limits the risk of exposure to COVID-19 and other viruses. The material’s non-biologic, fully synthetic construction also means that the matrix presents no risk of disease or viral transmission in contrast to donor tissue [7].

While the results presented in this case study are encouraging, this is only a small and preliminary case series with two patients and no control arm. The retrospective nature of the study means that the study was subject to the availability of the data records. However, the positive outcomes justify additional future work and further assessment to confirm the benefits of the synthetic hybrid-scale fiber matrix for the treatment of hematomas.

## Conclusions

Treatment of hematomas following the use of the synthetic hybrid-scale fiber matrix resulted in complete wound healing and was well-tolerated by the patients. Traumatic subcutaneous hematomas can be complex to treat, particularly in patients with multiple co-morbidities and on anticoagulants. In this study, hematomas of the foot and the leg demonstrated successful healing following treatment with the synthetic hybrid-scale fiber matrix.

Complete closure of all wounds was observed after two to three applications of the synthetic matrix within six to 16 weeks. The patients experienced no adverse events. The successful clinical application of the synthetic hybrid-scale fiber matrix suggests that this biomaterial may offer notable benefits as part of a new treatment paradigm for hematomas and warrants further investigation. 
